# Supplementary material for: Cooperation in the face of disaster
Source: PLoS One. 2025 Apr 3;20(4):e0318891. doi: 10.1371/journal.pone.0318891 (PMC11967933; doi:10.1371/journal.pone.0318891)
Supplement: S1 Appendix — This file contains the details of the experiment design and implementation. (PDF) [file pone.0318891.s001.pdf]

Materials and methods

The main experiments (*Control*, *10P*, *40P*, and *Level*) had participants totaling 884 individuals (221 groups), consisting both students and non-students, conducted separately at Stockholm University (440 individuals) and the University of the Philippines (444 individuals). Participants were recruited using recruitment posters and lab participants database at the Stockholm University's lab at the Centre for Cultural Evolution. Supplementary experiments were conducted in Sweden for the additional probability treatments (an additional 60 individuals for the 70% treatment and 64 individuals for the 100% treatment).

The study involved soliciting decisions of human participants. As such, we abided by the guidelines of the Swedish Ethical Review Authority, as well as the guidelines of the research ethics committee at the Stockholm School of Economics and the Cultural Evolution Lab at Stockholm University. Certification has been issued from the Stockholm School of Economics Ethical Committee and from the Swedish Ethical Review Authority that no ethical permit is required for the experiments conducted in this study. We also conducted the study according to the principles expressed in the All European Academies' (ALLEA) "European Code of Conduct for Research Integrity", which is endorsed by the Swedish Research Council.

The participants signed written consent forms in Swedish (in Sweden) and English (in the Philippines) prior to participating in the experiments. They were free to leave the experiment at any time without giving any reason. The consent forms were archived by the corresponding author. The participants were also given verbal orientation/introduction and written instructions prior to the experiment and were offered debriefing after the experiment. Lab personnel were available for their assistance and queries. The participants were all adults, and no minors were included. The study followed GDPR rules and did not gather personal or sensitive information. The experiment design did not entail any deception, and the data was analyzed anonymously. The experiments were conducted in computer lab settings, and made use of Behavery, an open-source cloud-based platform for behavioral experiments [1]. Full information was provided prior to the start of the experiment – instruction sheets were given to the participants, they answered control questions and did several trial rounds to ensure that they had full understanding of the instructions, and had access to lab personnel if they had queries.

The experiments were conducted during various periods in 2012-2013, involving 884 adult participants, in Sweden (September 2012; December 2012-January 2013) and the Philippines (August 2012; February 2013). The experiments were conducted at the game laboratory at the Centre for the Study of Cultural Evolution (CEK) at Stockholm University, and the computer laboratory of the Computational Science Research Center (CSRC) at the University of the Philippines, Diliman. Participants were recruited 2-3 weeks before the scheduled dates, through the laboratory's database in the form of e-mail sign-up invitation (i.e. Sweden), as well as through direct approach, signing-up, campus posters and social media announcements (i.e. Philippines). Several pilot studies were also conducted (2011-2012) in both countries to aid the design and detect practical and technical problems with the software, etc., but data in the pilot were not included in the study.

After the last round of the experiment, the participants answered a questionnaire, wherein among others, their emotions, risk preferences, motivations and comments were solicited. Emotions were elicited using the I-PANAS-SF measure (10 items), which

gauged the positive and negative affects or moods of the participants after the experiment [2]. The locus of control was measured through the Levenson's IPC Scale (24 items), which assessed if participants attributed events to internal, powerful others or chance factors [3]. Risk preference was elicited using a self-reported measure of general risk willingness, which largely offers a reliable indicator of one's risk behavior [4].

References

1. Funcke A. Behavery: Providing human and artificial behavior for experiments; 2015. `https://behavery.com`.

2. Thompson ER. Development and validation of an internationally reliable short-form of the positive and negative affect schedule (PANAS). Journal of cross-cultural psychology. 2007;38(2):227–242.

3. Levenson H. Differentiating among internality, powerful others, and chance. Research with the locus of control construct. 1981;1:15–66.

4. Dohmen T, Falk A, Huffman D, Sunde U, Schupp J, Wagner GG. Individual risk attitudes: Measurement, determinants, and behavioral consequences. Journal of the european economic association. 2011;9(3):522–550.
