# Supplementary material for: Cooperation in the face of disaster
Source: PLoS One. 2025 Apr 3;20(4):e0318891. doi: 10.1371/journal.pone.0318891 (PMC11967933; doi:10.1371/journal.pone.0318891)
Supplement: S2 Appendix — This file contains the Nash equilibrium analyses of the different experiment groups. (PDF) [file pone.0318891.s002.pdf]

Nash equilibrium analyses

In this section we will mathematically express the earnings (utility) of a player in a round in the different experiment categories, as described in the section Experiment design and theoretical predictions, and determine the Nash equilibria (NE).

Control group

The utility π_i for player i in a group of 4 players can be expressed in the following equation with endowment 20 and contributions $x_i \in \{0, 1, \dots, 20\}$ of player $i = 1, 2, 3, 4$.

$$\pi_i = \pi_i(x_1, x_2, x_3, x_4) = (20 - x_i) + \frac{1.6}{4} \sum_{k=1}^{4} x_k = 20 - 0.6x_i + 0.4 \sum_{k \neq i} x_k$$

The game is symmetric (since the utility π_i for player i to play x_i only depends on the sum of the contributions of the other players in the group, and not on any specific player), so we can express π_i as a function of x_i and g_i where $g_i = g_i(x_1, x_2, x_3, x_4) = \sum_{k \neq i} x_k$ is the sum of the contributions of the other players:

$$\pi_i = \pi_i(x_i, g_i) = 20 - 0.6x_i + 0.4g_i.$$

Since this is the earnings in each round in the control group as well as in the treatment groups when the threshold check passes, we will use it frequently, so for ease of notation let us introduce the function

$$c(x, g) = 20 - 0.6x + 0.4g.$$

To find the Nash equilibria, we will maximize π_i for

$$(x_i, g_i) \in \{0, 1, \dots, 20\} \times \{0, 1, \dots, 60\} =: S$$

Since π_i is linear in x_i and g_i, with x_i-coefficient $-0.6 < 0$ and g_i-coefficient $0.4 > 0$, the maximum of π_i is obtained in $(x_i, g_i) = (0, 60)$. Therefore, contributing 0 is always the best response to whatever the other players contribute, and the strategy (contribution profile) $(0, 0, 0, 0)$ is a Nash equilibrium. The function graph of the utility function π_i is visualized in Fig 1, where the best response (maximum of π_i) to each value of g_i is indicated with a black dot.

The 10P, 40P and Impact treatments

When we introduce a probability $p > 0$ for a check and the threshold 60 in each round, we get the following *expected* utility for *10P* where $p = 0.1$, and for *40P* where $p = 0.4$:

$$E\pi_i = \begin{cases} c(x_i, g_i) & \text{if } x_i + g_i \geq 60 \\ p \cdot (-M_i) + (1-p)c(x_1, g_i) & \text{if } x_i + g_i < 60 \end{cases}$$

where M_i is the accumulated earnings for player i. This is because a player loses the accumulated earnings M_i if there is a check (for which the probability is p) and the threshold is not met (i.e. $x_i + g_i < 60$). To make the one-shot game symmetric, we make the simplification that all $M_i = M$ are equal, i.e. that all members of a group risk to lose the same amount.

Assuming $M = 0$, the expected utility functions for *10P* and *40P* are visualized in Fig 1, where the best response (maximum of $E\pi_i$) to each value of g_i is indicated with a black dot. In *10P*, a shift in the best response occurs when the total contributions of the other group members is $g_i = 54$. The best option is to contribute zero unless the sum of the contributions of the other players is ≥ 54, in which case it is best to contribute 0 to 6 to exactly meet the threshold 60. In *40P*, the shift occurs at $g_i = 40$, so it is best to contribute zero unless the sum of the contributions of the other players is 40 and above, in which case it is best to contribute 0 to 20 to exactly meet the threshold.

In the *Impact* treatment, the following situation arises if there is a check and the threshold is not reached: both accounts are reduced to 0 with probability 1/3; only the individual account is reduced to 0 with probability 1/3; and only the group account is reduced to 0 with probability 1/3. Put in another way, the individual account is reduced to 0 with probability 2/3, and independently of this, the group account is reduced to 0 with probability 2/3. Thus, the expected utility in this treatment is

$$E\pi_i = \begin{cases} c(x_i, g_i) & \text{if } x_i + g_i \geq 60 \\ \frac{2}{3}p \cdot (-M_i) + (1 - \frac{2}{3}p)c(x_i, g_i) & \text{if } x_i + g_i < 60 \end{cases}.$$

If $x_i + g_i < 60$, i.e. the group *does not* reach the threshold 60, any contribution greater than 0 is suboptimal. This means that it is always better to give 0 because if there is a check, a player will lose her contribution, and if there is no check, we have a situation equivalent to the control group where the dominating strategy is to contribute 0.

Assuming $M_i = 0$, the expected utility function for *Impact* is visualized in Fig 1, where the best response (maximum of $E\pi_i$) to each value of g_i is indicated with a black dot. A shift in the best response occurs at $g_i = 47$, so it is better to contribute zero unless the sum of the contributions of the other players is 47 to 60, in which case it is best to contribute 0 to 13 to exactly meet the threshold.

This results in a (strict) Nash equilibrium of (0,0,0,0), where a contribution of $x_i = 0$ is the best response to $g_i = 0$. On the other hand, if the group *does* meet the threshold, $x_i + g_i \geq 60$, any player will be better off by lowering her contribution to make sure the threshold is exactly met ($x_i + g_i = 60$).

Thus, intuitively, any equilibrium must be either (0,0,0,0) or of the form (x_1, x_2, x_3, x_4) with $\sum_{k=1}^{4} x_k = 60$. More formally, for a fixed $g_i = g \in [0, 60]$, and assuming $M_i = M$ as before, the function

$$f(x_i) = \begin{cases} c(x_i, g) & \text{if } x_i + g \geq 60 \\ p \cdot (-M) + (1 - p)c(x_i, g) & \text{if } x_i + g < 60 \end{cases}$$

is linearly decreasing with an (increasing) jump at $x_i = 60 - g$. Therefore, the maximum of $f(x_i)$ is obtained either at $x_i = 0$ or $x_i = 60 - g$. In the case $g < 40$, we are always in the area $x_i + g < 60$, so the maximum of $f(x_i)$ is obtained at $x_i = 0$. In the case $g > 40$, we can be either in the area $x_i + g < 60$ or $x_i + g \geq 60$ so the maximum is obtained at $x_i = 0$ whenever $-pM + (1 - p)(20 + 0.4g) > 20 - 0.6(60 - g)$, which can be simplified to

$$g < \frac{36 - (M + 20)p}{0.6 + 0.4p} =: w(p, M)$$

and similarly, the maximum is obtained at

$$x_i = 60 - g \text{ whenever } g \geq w(p, M). \tag{1}$$

Since we already concluded that $(0, 0, 0, 0)$ is an equilibrium, let us look into the candidates $x_1 + x_2 + x_3 + x_4 = 60$. In order for (x_1, x_2, x_3, x_4) to be an equilibrium, by

Eq (1) we must have

$$x_i = 60 - \sum_{k \neq i} x_k \text{ and } \sum_{k \neq i} x_k \geq w(p, M)$$

for all $i = 1, 2, 3, 4$. Since $\sum_{k=1}^{4} x_k = 60$, this yields the system of equations

$$\begin{cases} x_1 + x_2 + x_3 + x_4 = 60 \\ x_1 \leq 60 - w(p, M) \text{ for all } i = 1, 2, 3, 4 \end{cases} \tag{2}$$

and we note that for different values of $w(p, M)$, Eq (2) has different sets of solutions.

Case 1: If $60 - w(p, M) < 15$, i.e. $w(p, M) > 45$, by the inequality in Eq (2), we must have $x_i < 15$, so $x_1 + x_2 + x_3 + x_4 < 60$, so Eq (2) has no solution.

Case 2: If $15 \leq 60 - w(p, M) < 16$, i.e. $44 < w(p, M) \leq 45$, by the inequality in Eq (2), we must have $x_i < 16$, so the only solution is $(x_1, x_2, x_3, x_4) = (15, 15, 15, 15)$.

Case 3: If $16 \leq 60 - w(p, M) < 17$, i.e. $43 < w(p, M) \leq 44$, by the inequality in Eq (2), we must have $x_i < 17$, so the solutions are

$$\left\{ (x_1, x_2, x_3, x_4) : 0 \leq x_i < 17 \text{ and } \textstyle\sum_{k=1}^{4} x_k = 60 \right\}$$

Computing for integer solutions, we find 35 Nash equilibria.

Case 4: If $17 \leq 60 - w(p, M) < 18$, i.e. $42 < w(p, M) \leq 43$, analogously to the above, the Nash equilibria consist of the 165 solutions

$$\left\{ (x_1, x_2, x_3, x_4) : 0 \leq x_i < 18 \text{ and } \textstyle\sum_{k=1}^{4} x_k = 60 \right\}.$$

Case 5: If $18 \leq 60 - w(p, M) < 19$, i.e. $41 < w(p, M) \leq 42$, analogously to the above, the Nash equilibria consist of the 445 solutions

$$\left\{ (x_1, x_2, x_3, x_4) : 0 \leq x_i < 19 \text{ and } \textstyle\sum_{k=1}^{4} x_k = 60 \right\}.$$

Case 6: If $19 \leq 60 - w(p, M) < 20$, i.e. $40 < w(p, M) \leq 41$, analogously to the above, the Nash equilibria consist of the 969 solutions

$$\left\{ (x_1, x_2, x_3, x_4) : 0 \leq x_i < 20 \text{ and } \textstyle\sum_{k=1}^{4} x_k = 60 \right\}$$

Case 7: If $60 - w(p, M) = 20$, i.e. $w(p, M) = 40$, analogously to the above, the Nash equilibria consist of the 1,771 solutions

$$\left\{ (x_1, x_2, x_3, x_4) : 0 \leq x_i < 20 \text{ and } \textstyle\sum_{k=1}^{4} x_k = 60 \right\}$$

Each of the cases 1-7 corresponds to a restriction on $w(p, M)$, generally of the form $l_1 < w(p, M) = \frac{36-(M+20)p}{0.6+0.4p} \leq l_2$. Solving for M in these inequalities gives

$$\frac{36 - 20p - l_2(0.6 + 0.4p)}{p} \leq M < \frac{36 - 20p - l_1(0.6 + 0.4p)}{p} \tag{3}$$

In the three scenarios $p = 0.1$ (*10P* treatment), $p = 0.4$ (*40P* treatment), and $p = \frac{2 \cdot 0.4}{3} = \frac{4}{15} \approx 0.27$ (*Impact* treatment), the limits on M, found by plugging in the appropriate values for l_1 and l_2 in each scenario into Eq (3), are summarized in Table 1, where * indicates that there are no (positive) values for M in which the case in question holds.

So, if M is high enough (i.e. more than or equal to 84 in *10P*, 9 in *Impact* and 0 in *40P*), all combinations that add up to 60 are equilibria. If it is low (less than or equal to 52 in *10P*, 0 in *40P* and 1.05 in *Impact*), the equilibrium is $(0, 0, 0, 0)$. In *40P*, no matter how much money you have in your account, all combinations of 60 are always in equilibria.

Case (number of NE in the cooperative regime)	*10P*	*Impact*	*40P*
1 (0)	$M < 52$	*	*
2 (1)	$52 \leq M < 58.4$	*	*
3 (35)	$58.4 \leq M < 64.8$	$M < 1.05$	*
4 (165)	$64.8 \leq M < 71.2$	$1.05 \leq M < 3.7$	*
5 (445)	$71.2 \leq M < 77.6$	$3.7 \leq M < 6.35$	*
6 (969)	$77.6 \leq M < 84$	$6.35 \leq M < 9$	*
7 (1,771)	$M \geq 84$	$M \geq 9$	Any M

Table 1. Limits on M.

Level treatment

Since the threshold in this case is either $50, 51, 52, \ldots, 70$ with each of these 21 possible values with equal probability $1/21$, we get the expected utility

$$E\pi_i = \begin{cases} c(x_i, g_i) & \text{if } x_i + g_i > 70 \\ p\left[\frac{x_i+g_i-49}{21}c(x_i,g_i) + \left(1 - \frac{x_i+g_i-49}{21}\right)(-M)\right] & \\ \qquad +(1-p)c(x_i,g_i) & \text{if } 50 \leq x_i + g_i \leq 70 \\ p(-M) + (1-p)c(x_i,g_i) & \text{if } 0 \leq x_i + g_i < 50 \end{cases}$$

The expected utility for a fixed $g_i = g \in [0, 60]$ can then be simplified to the following utility function, with $p = 0.4$:

$$f(x_i) = \begin{cases} c(x_i, g) & \text{if } x_i + g > 70 \\ \frac{1}{525}\Big(-6x_i^2 + (305 - 10M - 2g)x_i & \\ \qquad +(130g - 10Mg + 4g^2 - 3500 - 700M)\Big) & \text{if } 50 \leq x_i + g \leq 70 \\ p(-M) + (1-p)c(x_i,g) & \text{if } 0 \leq x_i + g < 50 \end{cases} \tag{4}$$

Assuming $M = 0$, the expected utility function for *Level* is visualized in Fig 1, where the best response (maximum of $f(x_i)$) to each value of g is indicated with a black dot. Here, the shifts occur at 44 and 54. This means that if the sum of the contribution of the other players is less than 44, it is best to contribute zero; if it is 44 to 53, the best is to contribute 17 or 18; if it is above 53, it is best to contribute 10 to 17 to make sure that the upper limit of 70 is exactly met.

In the following, let us call the three conditions in Eq (4) *regime 1* ($0 \leq x_i + g < 50$), *regime 2* ($50 \leq x_i + g \leq 70$), and *regime 3* ($x_i + g > 70$).

Case 1: If $g < 30$, we are always in regime 1, thus the best response to these g-values is $x = 0$.

Case 2: If $30 \leq g \leq 50$, the maximum of $f(x)$ is obtained either at $x = 0$ (regime 1) or somewhere in regime 2. Since $f(x)$ is quadratic in regime 2 with a negative quadratic coefficient $\left(-\frac{6}{525}\right)$, to find a possible local maximum in the interior of regime 2, we differentiate with respect to x and get

$$f'(x) = \frac{1}{525}(-12x + 305 + 10M - 2g).$$

So the local maximum situated at $x = (305 + 10M - 2g)/12$, whose smallest possible value in $30 \leq g \leq 50$ and $0 \leq M \leq 880$ is $x = (305 + 10 \cdot 0 - 2 \cdot 50)/12 \approx 17.1 > 17$. Thus, the local maximum is either obtained inside regime 2 ($x < 20$) or to the right of it ($x \geq 20$). Consider the two possibilities:

(i) The local maximum is obtained to the right of regime 2. Then the maximum of $f(x)$ must be obtained at $x = 20$. Thus, the only possible values for best responses in

this case are 0 and 20. The only candidates for NE are therefore (x_1, x_2, x_3, x_4) with $30 \leq x_1 + x_2 + x_3 + x_4 \leq 50$ and each $x_i \in \{0, 20\}$. The only such quadruple is $(20, 0, 0, 0)$ or a permutation thereof. However, none of these can constitute a NE, since the best response to $g = 0 + 0 + 0 + 0$ is $x = 0$, not $x = 20$.

(ii) The local maximum is obtained inside regime 2. This happens when $(305 + 10M - 2g)/12 < 20$, i.e. when $2g - 10M > 65$. But since $30 \leq g \leq 50$, we have $2g - 10M > 60 - 10M > 65$ and so we have an interior maximum only for $M < 0.5$.

Thus, for Case 2, the only NE are $(0, 0, 0, 0)$ (except for extremely small values of M – for example, when $M = 0$, $(17, 17, 17, 17)$ is an equilibrium).

Case 3: If $g > 50$, we need to compare the utility in regime 2 with that in regime 3. Under case 2 we established that the local maximum of the quadratic function in regime 2 is obtained at $x = (305 + 10M - 2g)/12$. Therefore, we have a local maximum in the interior of regime 2 iff $(305 + 10M - 2g)/12 < 20$ i.e. when $2g - 10M > 65$. But since $g > 50$, we have $2g - 10M > 100 - 10M > 65$, and so we have an interior maximum only for $M < 3.5$. So when $M \geq 3.5$, the maximum of f in regime 2 is obtained along the line $x + g = 70$. On the other hand, the maximum of f in regime 3 is also obtained along this line (because of the negative x-coefficient (-0.6). Thus, by symmetry, in this case, the NE are all 286 combinations of contributions adding up to 70:

$$\left\{(x_1, x_2, x_3, x_4) : 0 \leq x_i \leq 20 \text{ and } \sum_{k=1}^{4} x_k = 70\right\}$$

Thus, except for extremely small values of M ($M < 3.5$), the only NE in *Level* are $(0, 0, 0, 0)$ and the 286 combinations adding up to 70, as shown in Fig 1.

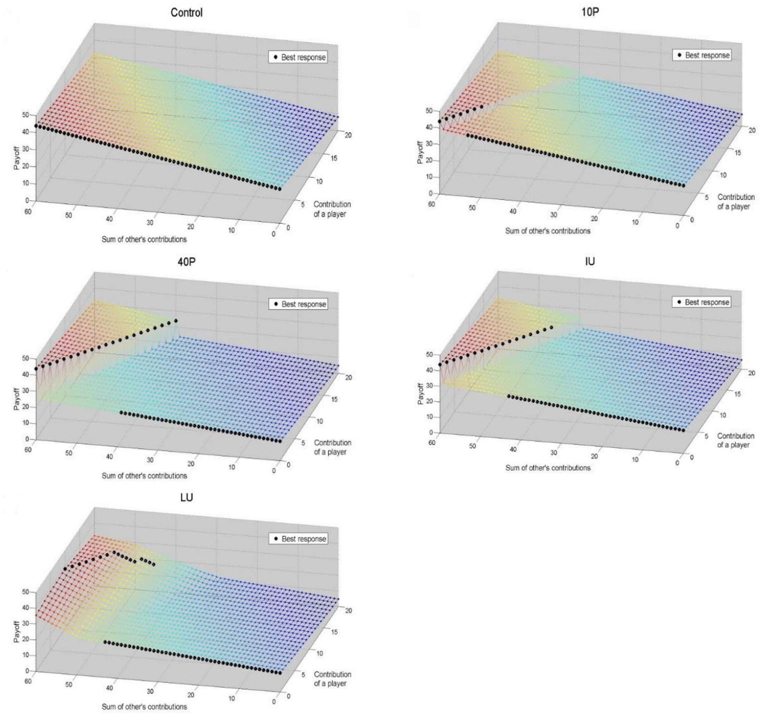

Fig 1. Expected utility functions for the experiment groups.
