# Supplementary material for: Cooperation in the face of disaster
Source: PLoS One. 2025 Apr 3;20(4):e0318891. doi: 10.1371/journal.pone.0318891 (PMC11967933; doi:10.1371/journal.pone.0318891)
Supplement: S3 Appendix — This file contains an analysis of how the participants conform to the rest of the group. (PDF) [file pone.0318891.s003.pdf]

Conformity tests

To additionally look into the conditional cooperation behavior, we looked into whether people increased, decreased or did not change their contribution, depending on if they were above, below or equal to the group average. In this method, people's contribution decisions are based on their beliefs on how they deviated from the group. Since the participants in our experiment also received information of the respective contributions of the other participants in the previous round anonymously, we extended the analysis to get a more nuanced results of conformity based on one's ranking in the group contribution. We wanted to see how multiple specific contributions and not just rough aggregate, would affect conforming behavior. To that end, we first ranked each contribution in the group according to size, with the largest contribution ranking as "1st" and the smallest as "4th". If the contributions in a group are 20-16-10-3, they will be ranked as 1-2-3-4. In accordance with the ranking algorithm in Wilcoxon's rank sum test, we assigned each observation in a tie its average rank. Thus, if the contributions in a group are 20-10-10-0, the ranks are 1-2.5-2.5-4. Secondly, we find that people conformed less in the treatments than in *Control*. Fig 1A shows the magnitude of the increase or decrease in contribution between two consecutive rounds in relation to one's contribution in the earlier round is above or below the average of the contribution of the other members. The adjustments are smaller in the treatments than in *Control*, reflecting less willingness to conform to others.

Adding more nuance by ranking the group members in terms of their contribution to the group, instead of the aggregate position of being above or below average, we find the biggest change in conformance was among those who gave most to the group (i.e. ranked first) and those who gave least to the group (i.e. ranked last). They did not decrease/increase their contributions in the Treatment as much as in *Control* (Fig 1B). This reduction in adjustment gives another evidence how the tendency to conform and is weakened in a stochastic environment.

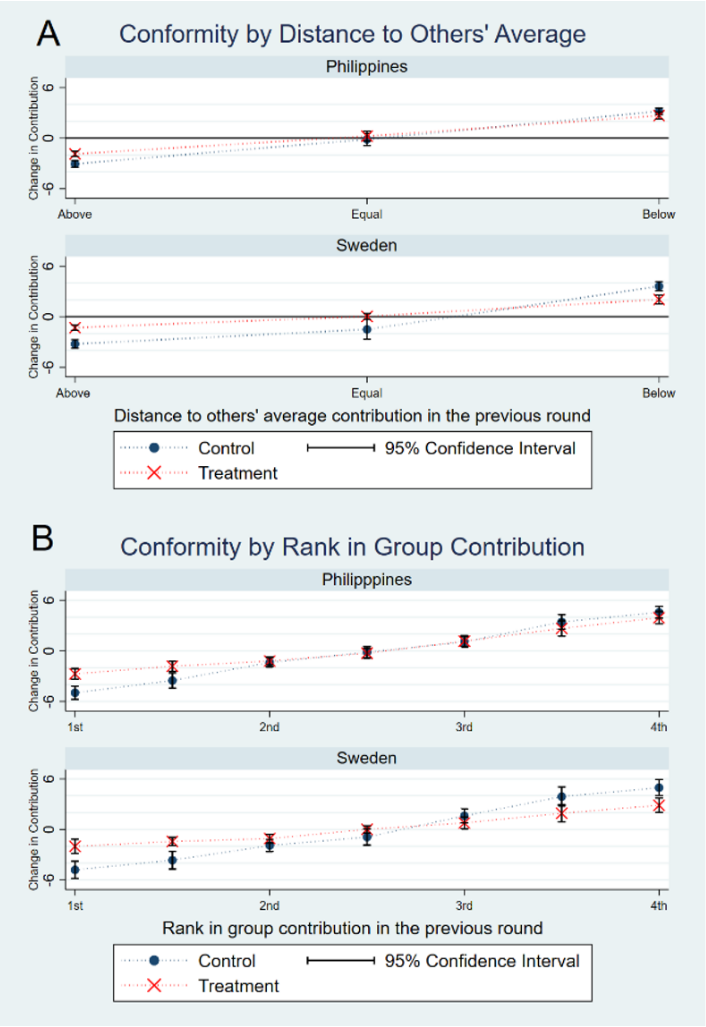

Fig 1. Results of conformity tests.
