# Supplementary material for: Cooperation in the face of disaster
Source: PLoS One. 2025 Apr 3;20(4):e0318891. doi: 10.1371/journal.pone.0318891 (PMC11967933; doi:10.1371/journal.pone.0318891)
Supplement: Attachment — Submitted filename: Rebuttal Letter for Revision.pdf [file pone.0318891.s005.pdf]

December 26, 2024

Dr. The Anh Han
Academic Editor
PLOS One

Re: Reply to reviewers comments for manuscript PONE-D-24-39170, "Cooperation in the Face of Disaster"

Dear Dr. Han,

We are very pleased to know that the two reviewers agree with you that the paper has good merit and addresses a highly relevant issue in behavioural evolution. Below, we provide a detailed point-by-point response to the reviewers' comments.

Responses to Reviewer #1

Comment 1: *"I think one of the key elements of this study is to test the three different types of uncertainty about the disaster (the probability of a disaster, threshold level, and impact). Therefore, I believe it is important to show the influences of them on the experimental performances. Although in Results, the authors mentioned contributions, contextual differences, responses to checks, and conditionality, the summary (figures) of results, especially the impact on the three different types of uncertainty, may be helpful for readers to understand."*

Response 1: The main result about the impact on cooperation of the different uncertainty types is captured by comparing contribution levels, which are clearly presented in Figure 2A and 2B. Thus, we are not sure that we correctly interpret this comment. But there are two results: (1) about the general effect on uncertainty compared to a certain environment, and (2) individual differences in contributions between the uncertainty types. To clarify this, we divided the Contributions section (under Experiment Results) into "Effect of uncertainty" (line 197) and "Effect of different uncertainty types" (line 212). Grouping the findings in these subsections can make it more helpful for readers to understand the results and figures.

Comment 2: *"The experiments were conducted during various periods in 2012–2013. I wonder why it took more than ten years to submit this manuscript. Would you explain the situation briefly?"*

Response 2: We appreciate this query regarding the timeline. The long gap between conducting the experiments and journal submission was due to a combination of circumstances. These are: change of research focus and job priorities, as well as incorporation of additional multimodal analyses, particularly the simulation models. This is to ensure robustness of the results and incorporate new perspectives to enhance the overall quality of the paper. We provide

transparency about this timeline in the Methods, and we can include this explanation if you and the editor think it is necessary.

Comment 3: *"Their simulation results support their experiments. Although the source code is available, I don't understand the detailed setting of the simulation. Please add the explanation."*

Response 3: We are not sure what is meant by "detailed setting", but we have added a description to the README-file in the GitHub repository where the simulation details are explained, as well as added code documentation to the source code.

Responses to Reviewer #2

Comment 1: *"In Abstract, the authors say that This is explained by a contribution divergence,' but I do not agree. Contribution divergence' is not an explanation, but just another way of describing the observation."*

Response 1: Thank you for this observation and we agree that "contribution divergence" is not an explanation but another way of describing the observation. We revised the Abstract, changing "This is explained by" to "In other words, we observe" a contribution divergence (line 14).

Comment 2: *"The authors have identified a player's utility with the earnings (line 463). However, when describing the experimental results, they mention risk-willingness (line 232), which implies that a player's utility could depend on his or her risk attitude. I guess that even their main observation that people become more favorable to cooperation in an uncertain environment could be described better by incorporating the risk attitude in utility properly."*

Response 2: We appreciate this comment. Game-theoretically, the utility consists of the monetary earnings alone. However, the risk-willingness naturally affects cooperation in uncertain/risky environments. To incorporate and clarify this, we added the following to the Contributions section under Experimental results (lines 206-211):

"That contributions are higher when there is a risk of losing earnings (unless a contribution threshold is met) is both intuitive and consistent with the Nash equilibrium analysis in the Materials and methods section. However, the extent to which contributions increase in the presence of a disaster risk naturally depends on the participant's risk-willingness. We see evidence of this in the Contextual differences section below."

This is made clear in the Philippines-Sweden comparison where Filipinos had a higher level of self-reported risk willingness, and thus also contributed less than Swedish participants. Making the player's utility depend on individual risk-willingness will make game-theoretical predictions complicated and will also require arbitrary assumptions on *how* risk-willingness affects utility.

In addition to addressing the reviewers' comments, other measures were also undertaken to comply with the PLOS ONE submission guidelines, as specified in the cover letter. Furthermore, we fixed the following typographical errors:

- Line 43: "cooperatove" to "cooperative"

- Line 57: “adressing” to “addressing”
- Line 102: “homogenous” to “homogeneous”
- Line 143: “experiement” to “experiment”
- Line 270: “perticipant’s” to “participant’s”
- Line 430: “commitee” to “committee”

We hope that the revisions adequately address all points raised by the reviewers and have improved the manuscript to fully meet PLOS ONE’s publication criteria. Thank you again for this opportunity to revise our work.

Sincerely,

Marijane Luistro Jonsson

Markus Jonsson
